# Editorial: Trust in Automated Vehicles

**DOI:** 10.3389/fpsyg.2024.1404200

**Published:** 2024-06-10

**Authors:** Francesco Walker, Yannick Forster, Sebastian Hergeth, Johannes Kraus, William Payre, Philipp Wintersberger, Marieke Martens

**Affiliations:** ^1^Cognitive Psychology, Leiden University, Leiden, Netherlands; ^2^BMW Group, Munich, Germany; ^3^Johannes Gutenberg University Mainz, Mainz, Germany; ^4^Coventry University, Coventry, United Kingdom; ^5^TU Wien, Vienna, Austria; ^6^University of Applied Sciences Upper Austria, Hagenberg, Austria; ^7^Industrial Design, Eindhoven University of Technology, Eindhoven, Netherlands

**Keywords:** trust, automated vehicles (AV), trust in automation, SAE levels, self-driving, trust calibration, automated driving, human machine interface (HMI)

## Introduction

Automated vehicles (AVs) promise to change the future of transportation, offering unprecedented benefits, from increased safety to enhanced efficiency. However, the realization of these advantages depends on user trust in the technology. While there is widespread agreement on the pivotal role of trust, numerous questions persist. In this Research Topic, we present 15 studies addressing some of the most important issues, in particular:

1) What is trust and how should we measure it?
2) How does trust affect in-vehicle user responses?
3) How do Human Machine Interfaces (HMIs) affect trust toward AVs?
4) How can we improve interactions between AVs and pedestrians?

## What is trust and how should we measure it?

*Trust in automated vehicles* is heavily discussed in the literature. Yet, there is a general lack of agreement on how trust should be conceptualized, calibrated and measured. Walker et al. provide an expert perspective on this theme, emphasizing the importance of experience. The article points out that good calibration of trust requires experience in a broad range of scenarios including system malfunctions. It goes on to propose a conceptual framework for understanding the development of trust in a particular automated driving system.

Of key importance in developing a good understanding of trust are the methods, measures, and approaches used to operationalize the concept. Walker et al., point out that many of the methods currently used to quantify trust lack reliability. Alsaid et al. focus on one such method, namely the use of trust questionnaires. In their study, they use text analysis to compare and contrast commonly used trust questionnaires and describe a Web application based on their methods. This kind of study can help researchers to select the trust questionnaires most appropriate for their research objectives. However, questionnaires should always be complemented with more dynamic measures capable of capturing short-term changes in drivers' dynamic learned trust. One possibility, proposed by Payre et al., is to use driver engagement in secondary tasks during automated driving as a surrogate measure for trust. Evidence presented by Nordhoff et al. supports this proposal.

Ultimately, what the community needs are more naturalistic on-road studies, focusing not just on individual drivers but on the way trust develops within groups facing shared risks and uncertainties. This is the approach taken by Momen et al., who analyze the conversations of groups of participants during rides in a Tesla Model X with Level 2 automated functions.

## How does trust affect in-vehicle user responses?

Trust strongly affects the way users interact with AVs. This is apparent in the survey by Nordhoff et al.. Respondents report that they rarely disengage partial automation due to boredom or sleepiness. However, they disengage more frequently when they do not trust the automated system. Aligning with this perspective, Payre et al. explore the impact of “screen failures” on user engagement in secondary tasks during conditionally automated driving (CAD). According to the authors, such failures caused by a cyberattack led to users taking control of the vehicle, negatively impacting the following manual driving performance. By contrast, Britten et al.'s Wizard-of-Oz study reveals that when a CAD system safely executes automated evasive maneuvers, users rarely intervene—a sign of high trust. In the same spirit, Taylor et al. show that drivers comply with takeover request (TOR) and have shorter takeover times when the behavior of the CAD vehicle is highly reliable (compared to a low-reliability condition).

In Taylor et al.'s study, the TOR is delivered by an in-vehicle agent. The study by Zieger et al. suggests that trust and take-over performance are influenced by the combined effect of the reliability of such agents, and the user's emotional state. Another dimension of trust is the familiarity of the systems drivers are meant to trust. Findings from Hunter et al. suggest that trust develops in similar ways in familiar systems (e.g., a car) and less familiar modes of transport (e.g., an unconventional form of “sidewalk mobility”).

Taken together, these studies highlight the interplay between trust, system reliability and user behavior, providing valuable insights that extend beyond individual trust dynamics to implications for the design of emerging mobility solutions.

## How do HMIs affect trust toward AVs?

Crucial information for drivers is typically communicated through HMIs. However, the effectiveness of existing HMIs is unclear, particularly in conditions when users need to keep their attention on the road and in situations requiring abrupt minimal risk maneuvers (MRMs).

Monsaingeon et al. demonstrate that multimodal interfaces, indicating the limitations of specific Level 2 automated driving systems, can promote an appropriate level of attention, increased mode awareness, and enhanced trust in automation. However, the observed effects are context-dependent. Worryingly, an improved understanding of the system's functioning does not always translate into improvements in observed driving performance.

Hub et al. propose that warning signs and 360° LED light bands displayed by the external HMI (eHMI) of an AV can enhance trust among following drivers, reducing the perceived criticality of MRM maneuvers. el Jouhri et al. study the impact of a color-themed HMI. Their results show that users perceived the color-themed version as more trustworthy and pleasant than a baseline HMI, leading to faster takeover reaction times.

Collectively, these findings highlight the importance of effective communication strategies for the future of AV technology.

## How can we improve interactions between AVs and pedestrians?

Automated vehicles need to earn the trust of all road users, with pedestrians standing out as a crucial demographic. Yet, it is challenging to communicate with non-drivers in ways that are efficient, comfortable, and easily comprehensible. Crucial information is typically conveyed through eHMIs. In their article, Bonneviot et al. show that eHMIs can enhance trust and increase pedestrians' willingness to cross the road in front of AVs. Notably, the study reveals that the use of anthropomorphic features in eHMIs leads to higher levels of trust and safer crossing behavior compared to conventional road signals. Bellet et al. demonstrate that perceptions and assessment of AV-yielding behaviors are mediated by age. They also show that well-designed eHMIs can help older people to assess the behavior of AVs more accurately, achieving safety judgments that align closely with those of younger participants.

## Conclusion

The studies in this Research Topic illustrate some of the key factors that shape user trust in and interactions with AV technology. The studies suggest ways of measuring trust and conceptualizing the development of trust over time. The findings show that trust calibration involves user experience in an ever-growing range of scenarios, and is strongly influenced by system malfunctions and reliability. Other important influences include the role of in-vehicle agents, HMIs, and the external appearance of AVs. The end results include improved attention and takeover performance, as well as safer interactions with other road users, especially the more vulnerable ones (e.g., pedestrians and the elderly). It is our firm belief that these results can provide valuable guidance for future research and the integration of AVs into our rapidly evolving mobility landscape.

## Author contributions

FW: Writing – review & editing, Writing – original draft, Project administration, Funding acquisition, Conceptualization. YF: Writing – review & editing. SH: Writing – review & editing. JK: Writing – review & editing. WP: Writing – review & editing. PW: Writing – review & editing. MM: Writing – review & editing.

